# Current Status and Future Development of Cell Transplantation Therapy for Periodontal Tissue Regeneration

**DOI:** 10.1155/2012/307024

**Published:** 2012-01-16

**Authors:** Toshiyuki Yoshida, Kaoru Washio, Takanori Iwata, Teruo Okano, Isao Ishikawa

**Affiliations:** Institute of Advanced Biomedical Engineering and Science, Tokyo Women's Medical University, 8-1 Kawata-cho, Shinjuku-ku, Tokyo 162-8666, Japan

## Abstract

It has been shown that stem cell transplantation can regenerate periodontal tissue, and several clinical trials involving transplantation of stem cells into human patients have already begun or are in preparation. However, stem cell transplantation therapy is a new technology, and the events following transplantation are poorly understood. Several studies have reported side effects and potential risks associated with stem cell transplantation therapy. To protect patients from such risks, governments have placed regulations on stem cell transplantation therapies. It is important for the clinicians to understand the relevant risks and governmental regulations. This paper describes the ongoing clinical studies, basic research, risks, and governmental controls related to stem cell transplantation therapy. Then, one clinical study is introduced as an example of a government-approved periodontal cell transplantation therapy.

## 1. Introduction

Since the 1980s, periodontal ligament cells have been considered a reliable source for periodontal regeneration. Nyman et al. reported that periodontal ligament (PDL) tissue possesses periodontal regenerative properties [1]. Based on this regenerative concept, several procedures have been introduced for the selective proliferation of PDL stem cells, such as guided tissue regeneration and enamel matrix derivative [2]. However, the desired regenerative outcomes have not been attained, especially for patients with severe periodontal defects. Experimental approaches to overcome the limitations of existing therapies have included the ex vivo expansion of stem cells derived from PDL, bone marrow, adipose tissue, and alveolar periosteum for transplantation as stem cell replacement therapy in animal studies. These studies have indicated that the transplantation of stem cells can be an effective treatment for periodontal defects [2]. As a consequence of these successful animal studies, the clinical application of stem cells for the regeneration of periodontal tissue has begun. However, the efficacy and safety of such cell-based therapies have not been fully evaluated, and the risks of stem cell therapies have been underscored by several clinicians and researchers. In this paper, we first review the current research targeting cell-based therapies for periodontal regeneration and then discuss the risks and governmental controls of stem cell transplantation therapy. Last, we will introduce our ongoing clinical study that was approved by the regulatory authority of the Japanese government.

## 2. Current Progress in Periodontal Cell Transplantation Therapy

### 2.1. Periodontal Ligament-Derived Mesenchymal Stem Cells (PDL-MSCs)

**Figure 1:**
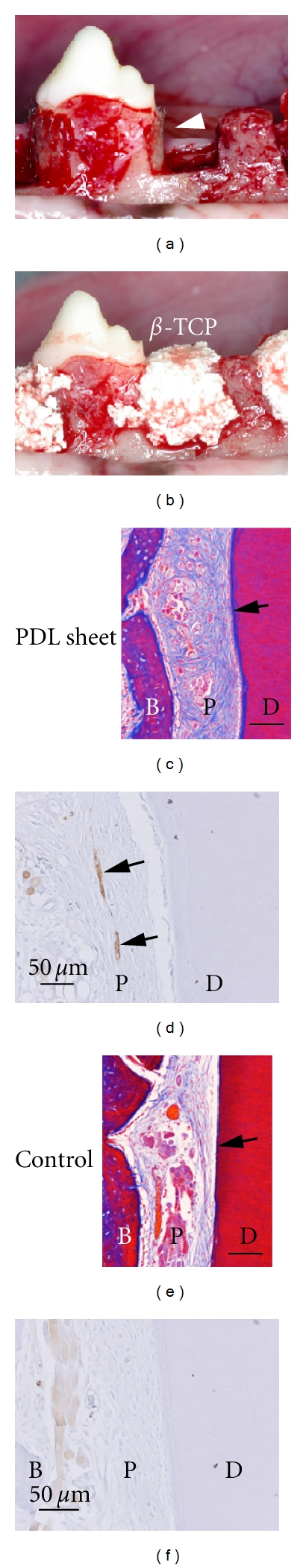
Periodontal tissue regeneration using PDL cell sheets. (a) Sheets of polyglycolic acid (arrowhead) with or without the cell sheets were applied onto the root surfaces of canine mandibular premolars. (b) Bone defects were filled with *β*-TCP. (c, e) Thick, newly formed cementum (arrow) and dense collagen fibres were observed in the PDL of the PDL sheet-transplanted group. (d, f) The immunohistochemical detection of neurofilament protein revealed that newly formed nerve fibres were only observed in the group given the PDL sheets (arrow). Scale bars: 100 *μ*m in (c) and (e); 50 *μ*m in (d) and (f). B: newly formed bone, P: periodontal ligament, and D: root dentin. Modified from [13] with permission.

Previous studies that reported the regenerative properties of PDL using animal models indicated the existence of stem cells in PDL tissue [3, 4]. Liu et al. reported that autologous PDL-MSCs enhanced regeneration of periodontal tissue, including alveolar bone, cementum and PDL in a minipig [5]. Feng et al. transplanted autologous PDL progenitors to three patients who suffered from periodontal disease. The results showed periodontal regeneration with no adverse effect [6]. Tissue engineering techniques have been applied to improve these cell-based therapies. Okano et al. developed a temperature-responsive cell culture technique to permit the harvest of adherent cultured cells by simply lowering the temperature [7, 8]. Our group produced PDL-derived cell sheets using this temperature-responsive culture dish and found that the cell sheets had a potential to promote regeneration of periodontal tissue, which was composed of bone, cementum, and PDL, in vivo (Figure 1) [9–12].

Allogeneic transplantation of PDL-MSCs into bone defects in minipigs has been shown to result in periodontal regeneration without significant immunological rejection [5]. It has also been shown that porcine PDL-MSCs possess a low immunogenicity and immunosuppressive function [14]. These data could shed light on the potential of allogeneic transplantations using PDL-MSCs at the clinical level and, thus, broaden the range of opportunities for cell transplantation therapy.

### 2.2. Periosteal Cells

Periosteal cells have been reported to be a potential source of cells for the regeneration of periodontal tissue [15, 16]. Recently, Mizuno et al. reported that cultured autologous periosteal cell membranes induced regeneration of periodontal tissues including bone, cementum, and periodontal ligament in a canine model of a class III furcation defect [17]. Following the results of these studies, clinical trials for cell transplantation therapy using periosteal cells were conducted. Human periosteal cell sheets with platelet-rich plasma (PRP) and hydroxyapatite (HA) were transplanted into 30 patients who suffered from chronic periodontitis, and this treatment was found to enhance periodontal regeneration [18].

### 2.3. Bone-Marrow-Derived Mesenchymal Stem Cells (BM-MSCs)

Bone-marrow-derived mesenchymal stem cells (BM-MSCs) have the potential to differentiate into various types of tissue, including bone, cartilage, adipose, muscle, and periodontal tissue [19–22]. Recent in vivo studies have shown that BM-MSCs could induce periodontal regeneration [21, 23]. Clinical trials using BM-MSCs with PRP have also been conducted, and the results indicated that periodontal regeneration could be induced by this approach as well [24].

### 2.4. Adipose-Derived Stem Cells (ADSCs)

Adipose-derived stem cells (ADSCs) are a useful source for cell transplantation therapy because this tissue is abundant and easy to obtain compared with other sources. ADSCs have been shown to be capable of differentiating into various tissue types [26–29]. It is reported that ADSCs mixed with PRP had the potential to regenerate periodontal defects in vivo [30–32], which supports the use of ADSCs in cell transplantation therapy for periodontal regeneration.

### 2.5. Gingival Fibroblast

To recover gingival recession, a cell transplantation therapy using gingival fibroblasts has been developed. For this technique, gingival fibroblast sheets were obtained by culturing gingival fibroblasts on collagen sponges [33]. The autologous gingival fibroblast sheets were then transplanted to 14 sites in 4 patients suffering from periodontal disease in order to achieve root coverage. The results demonstrated regeneration of gingival tissue and suggested that gingival fibroblasts could be readily harvested and prepared for transplantation [34].

### 2.6. ES/iPS Cells

Because embryonic stem (ES) cells and induced pluripotent (iPS) cells can differentiate into all types of somatic cells, so-called pluripotency, they are thought to be the ultimate cellular source for regenerative therapies. Recently, clinical trials using ES cells have begun in the United States. However, tumorigenesis is a common concern with this application of ES/iPS cells. Thus, it is highly desired to achieve commitment of the ES/iPS cells to a differentiated state prior to transfer. Tada et al. demonstrated the differentiation of ES/iPS cells into a mesodermal lineage [35] and Duann et al. demonstrated alveolar bone regeneration by iPS cells in an animal model [36]. Furthermore, recent reports have indicated the sparse existence of pluripotent cells among MSCs [37, 38], which expands the possibilities for the use of pluripotent cells in cell transplantation therapy. However, little is known about the mechanisms and pathways that control the differentiation. Further studies are therefore needed in regard to periodontal regenerative therapy.

## 3. Characteristics and Future of MSC

Because the PDL, bone marrow, adipose, and periosteal tissues are thought to contain MSCs, cells from these tissues can be used to regenerate mesenchymal tissue, including alveolar bone and periodontal ligament. MSCs were first identified from bone marrow as plastic-adherent fibroblastic cells and shown to possess a multipotency to differentiate into multiple mesenchymal lineages, such as osteocytes, chondrocytes, and adipocytes [39, 40]. As early as 1995, MSCs from multiple sources were transplanted into patients because of their proliferative property, immunomodulatory, and trophic effects [41].

Cells extracted enzymatically from tissue tend to possess proliferative capacities and multipotency. In the dental field, the multipotency of dental pulp cells was first described by Gronthos et al. [42]. This group introduced a method for digesting tissue with enzymes that dramatically expanded stem cell research in the dental field. Compared to conventional outgrowth methods, enzyme treatment allows for the harvest of a variety of cell types, such as fibroblastic cells, vascular cells, and nerve cells, among others [42]. Furthermore, the characteristics of these cells have been shown to become homogenised during cultivation.

For clinical considerations, each type of MSC has advantages and disadvantages. PDL-MSCs have been shown to exhibit a greater regenerative capacity for PDL tissue compared to BM-MSCs and periosteal cells in canine one wall defect, model for severe periodontal defect [13]. Because clinicians routinely extract teeth, autologous PDL-MSCs can easily be obtained from patients. However, PDL-MSCs cannot be applied to patients who lack teeth to be extracted. Periosteal cells are more easily and abundantly harvested than PDL-MSCs and BM-MSCs, although periosteal cells require a longer culture period to obtain a sufficient number of cells for transplantation as compared to PDL-MSCs [17, 43]. BM-MSCs have been transplanted into many patients in various clinical studies and are thought to be suitable for cell transplantation therapy due to their multipotency and high cellular proliferation rate. However, the collection of bone marrow requires patients hospitalised. Although there are many data from animals and cells study, there is not enough clinical data to compare the efficacy of each type of MSC and the efficacy on severe periodontal defect including horizontal defect. Thus, it is currently difficult to draw conclusions about which MSC is most suitable for human periodontal regenerative therapy.

Recent studies have revealed that MSCs can be extracted from other tissues. One group of researchers demonstrated that MSCs are localised in the perivascular compartment [44]. Park et al. reported that PDL-MSCs could also be obtained from inflamed PDL tissue [45]. Because inflamed PDL tissue can be collected during periodontal treatment, it is possible that this technology will broaden the range of indications for cell transplantation therapy using PDL-MSCs.

## 4. Guarantees of Safety and Efficacy for Stem Cell Transplantation Therapy

Stem cell transplantation therapy is a promising technology that can regenerate periodontal tissue. However, the efficacy and safety of cell transplantation therapies are not well understood. There have been several clinical reports and reviews published that describe the risks and side effects of stem cell therapies. To protect patients from such risks, regulatory bodies in several countries have begun to establish guidelines and regulations for the control of stem cell therapies. Therefore, it is imperative that clinicians who aim to undertake stem cell therapies understand the associated risks and regulations. In the following section, we provide an overview of the risks related to stem cell therapies and then describe our work on a clinical study that was approved by the regulatory authorities of the Japanese government.

## 5. Risks of Stem Cell Transplantation Therapy

Many scientists suppose and indicate the risks of stem cell transplantation [46–48]. Indeed, severe side effects from stem cell therapies have been reported. One major concern about stem cell transplantation therapy is the tumorigenic property of the stem cells themselves. Notably, the formation of a transplanted cell-derived brain tumour in a patient who received neural stem cell transplantation was reported [49], although the precise mechanisms responsible for the formation of this tumour remain unclear. For MSC-based transplantation, no tumour formation has been reported during the course of clinical studies involving periodontal cell therapies [46, 50]. However, because the tumorigenic potentials of cells are thought to vary by cell type and application site, the risk of tumour formation must be investigated in the context of each type of cell transplantation therapy.

Other risks associated with stem cell transplantation therapy have been suggested, including those associated with cell harvesting, cell culture, the site of administration, and the interaction between the transplanted cells and the recipient's immune system [48, 51]. Because stem cell transplantation therapy is a new type of treatment, there are few published data on the fate of stem cells after transplantation. Therefore, it is important to confirm the safety of these approaches before applying them to patients, and all data related to the clinical course of each cell transplantation therapy should be recorded. As a result, these strategies should help to reduce patient risk.

## 6. Governmental Control over Cell Transplantation Therapy

In recent years, many new cell therapies have been developed, and their numbers continue to grow. However, there are many cell therapies that are inadequately designed, lacking evidences of efficacy, safety, and patient protection. For the protection of patients from such cell therapies, the governing bodies of Japan, the European Union, and the United States have been establishing regulations over the control of the quality of cell transplantation therapies based on Good Clinical Practice (GCP) and Good Manufacturing Practice (GMP).

GCP is an international ethical and scientific quality standard that applies to clinical studies and governs their design and patient protection [52]. According to GCP, data and plans, including the evidence, potential risks and benefits of the cell transplantation therapy, the protection of patients' rights, the scientific propriety of the protocol, and data management, must be approved by the institutional review board and independent ethics committee. GMP governs the cell culture processes for cell transplantation therapy [53]. GMP requires that the materials, protocols, tools, and environments used for cell transplantation therapy are guaranteed as safe and effective. GMP also requires a record of all cell culture procedures to be kept in the laboratory for traceability.

Conforming to these regulations requires significant amounts of work and carries a high cost [54]. Cell transplantation therapy under such regulations are often too expensive and too late for patients who are in critical need. Therefore, existing mainstream regulations are sometimes criticised as overly strict and repressive. Indeed, patients who wish to receive cell transplantation therapy have been known to travel to countries where little or no regulation of these procedures is required to receive the therapy; this is referred to as “stem cell tourism” [54–56]. However, the safety and efficacy of many of these therapies have either been questioned or entirely invalidated. Several such cell therapies have been shown to exhibit no effect, and several patients have died from diseases or complications resulting from the cell transplantations [55, 57–61]. It is therefore important for clinicians to implement cell transplantation therapies only after their efficacy and safety have been validated.

## 7. Preparation for Clinical Trial

Our clinical study of periodontal regenerative therapy using cell sheet technology has been prepared according to the guidelines of the Japanese Ministry of Health, Labour, and Welfare. As we previously demonstrated that PDL cell sheets were effective for periodontal tissue regeneration in animal models [9, 11, 62], we have prepared for clinical trial with this transfer approach. The methods of autologous cell transplantation and cell culture with autologous serum were selected to avoid potential infections from animals or other people. To reduce the risk of tumour formation and to enhance hard tissue formation, the cells were cultured in medium that promoted osteoblastic differentiation after they had been seeded onto temperature-responsive culture dishes.

We optimised a protocol for harvesting human PDL cells that would retain their proliferative, osteogenic, and multipotency [43]. Regarding the validation of the quality of the cell sheets, we found that periostin and alkaline phosphatase (ALP) could be used as quality-control markers of PDL and osteoblast differentiation, respectively [43]. Next, using a canine model for a preclinical study, we investigated the periodontal regenerative capacity of cell sheets that were prepared according to this protocol. The results indicated that the combined use of autologous PDL cell sheets and *β*-tricalcium phosphate was able to regenerate alveolar bone and periodontal ligament tissue at defective sites [12].

**Figure 2:**
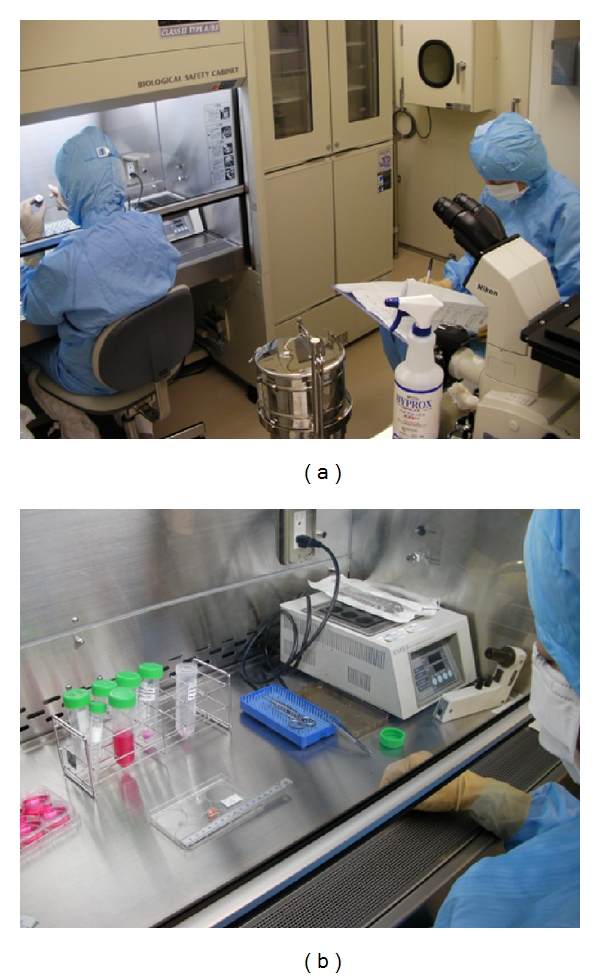
CPC of Tokyo Women's Medical University. (a) Individuals who work in the CPC wear sterile clothing, gloves, caps, and masks to avoid releasing possible contaminants. (b) All tubes and dishes were labelled to avoid misidentification.

We also established a culture protocol for the preparation of cell sheets that can be applied to patients. PDL cell culture was performed in a clean suite known as the Cell Processing Center (CPC). The CPC is composed of several rooms with controlled air flow in addition to separated personnel and material line of flow to maintain the cleanliness of the room and to prevent any possible contamination between personnel and cells [63]. All of the work in the CPC was carried out according to the Standard Operating Procedure (SOP), a cell culture working protocol that ensures the quality of the cell sheets. The work in the CPC was always performed by 2 individuals; one performed the culture procedures and the other directed the procedures to avoid human error and to keep a record of all of the procedures (Figure 2) [63].

**Figure 3:**
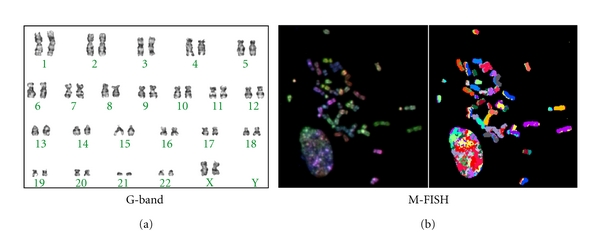
*Karyotype of cultured human PDL cells*. (a) G-banding staining revealed that the number, banding, and shape of the chromosomes were normal. (b) Multiplex fluorescence in situ hybridization (M-FISH) that visualises each chromosome in a different colour showed that no chromosomal aberrations, including translocation, had occurred.

**Figure 4:**
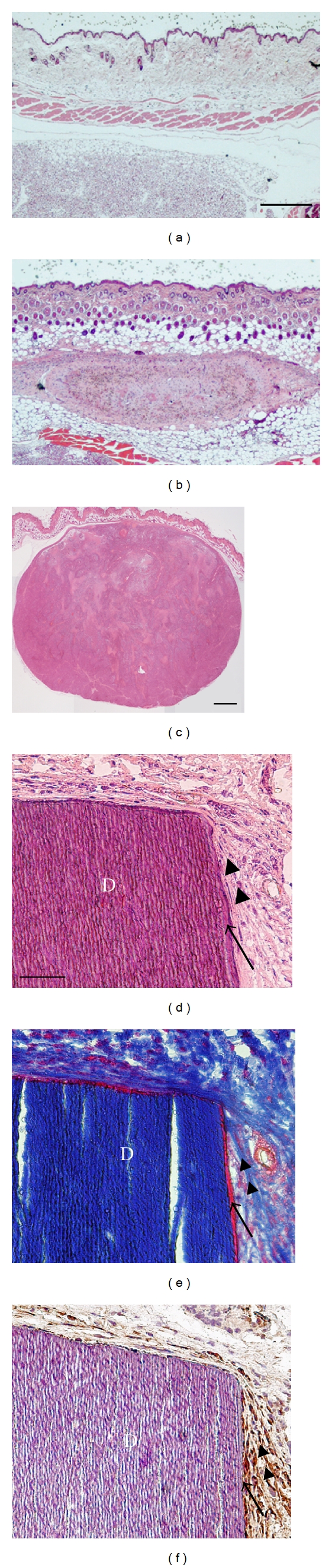
Tumorigenic and regenerative properties of human PDL cell sheets. (a)–(c) Tumorigenesis in vivo. Immunodeficient mice were injected with medium only (negative control), human PDL cells, or HeLa S3 cells (positive control). Mice injected with human PDL cells or the medium control did not form tumour-like tissue (a, b), whereas mice injected with HeLa S3 cells exhibited tumour formation (c). (d)–(f) Histology and immunohistochemistry of implanted human PDL cell sheets with dentin blocks. Newly formed cementum- (arrow) and PDL-like tissue (arrowheads) were observed around the dentin blocks (d, e). Immunostaining with an antibody against human vimentin revealed the presence of human PDL cells around the dentin block (f). Scale bars: 500 *μ*m in (a) and (b); 1 mm in (c); 50 *μ*m in (d)–(f). Modified from [25] with permission.

After establishing this SOP, we investigated the safety of the cell sheets that were prepared in this manner. We demonstrated that the cell sheets were free of contamination by bacteria or mycoplasma [25]. Because several studies had indicated that transformation of cells occurred during culture [64, 65], both in vitro and in vivo tumorigenicity tests were carried out [25]. Karyotype testing revealed that no chromosomal abnormalities related to tumorigenicity or other diseases had occurred in the cultured human PDL cells (Figure 3). By the soft agar test, cells from the PDL cell sheets exhibited no evidence of tumorigenic potential in vitro. Furthermore, the injection of cells isolated from PDL cell sheets into immunodeficient mice caused no tumour formation, whereas the injection of cancer cells reliably caused tumours to form (Figure 4) [25].

Next, we confirmed the in vivo and in vitro regenerative properties of the cell sheets that had been prepared according the SOP. For in vitro analysis, the cell sheets were found to be composed of adequate numbers of living cells that expressed periostin and ALP [25]. We then transplanted cell sheets with dentin blocks into immunodeficient mice and confirmed the formation of PDL- and cementum-like tissue around the dentin block (Figure 4) [25]. We also prepared a document that contained a full record of these procedures and the resulting data for submission to the Japanese Ministry of Health, Labour, and Welfare. This clinical study of PDL cell sheet transplantation was approved in January 2011.

As previously mentioned, the generation of cell transplantation materials according to the specified regulations is an enormously expensive and time-consuming process. For example, the CPC cost 2 million US dollars to build and 15,000 US dollars per year to maintain [63]. The performance of the safety and efficacy tests and the preparation of the necessary paperwork also represent significant burdens for the clinicians and associated university staff. Nevertheless, we believe that governmentally regulated cell transplantation therapy remains the best option for patients. One clinical solution may be the formation of collaborations between academic medical centres with existing CPCs. In this way, the outside clinician would collect tissue from a patient and send it to the university, where the cells could be harvested and cultured in a CPC and then sent back to the clinician for the cell transplantation therapy. For this purpose, Nozaki et al. developed a special container capable of maintaining an internal temperature of 37°C for more than 30 hours without a power supply. Using this container, cell sheets could be harvested without losing viability after 8 hours of transportation [66]. Another solution may be the use of closed or automated cell culture systems. There are several GMP-compliant automated cell culture systems currently available. Although the necessary equipment is expensive, the total cost would probably be far less than that of building and maintaining a CPC.

## 8. Conclusion

In this paper, we have provided an overview of the state of periodontal regenerative therapy using stem cells. Many patients suffer from periodontitis, and clinicians have struggled to regenerate the lost alveolar bone. Stem cell therapy is a promising nascent therapy that may allow the regeneration of lost periodontal tissue. Although there are many issues that need to be resolved before stem cell therapies become common, clinicians should continue to keep a watchful eye on the progression.
